# Application of machine learning with large-scale data for an effective vaccination against classical swine fever for wild boar in Japan

**DOI:** 10.1038/s41598-024-55828-6

**Published:** 2024-03-04

**Authors:** Satoshi Ito, Cecilia Aguilar-Vega, Jaime Bosch, Norikazu Isoda, José Manuel Sánchez-Vizcaíno

**Affiliations:** 1 VISAVET Health Surveillance Center, Complutense University of Madrid, Madrid, Spain; 2 Department of Animal Health, Faculty of Veterinary, Complutense University of Madrid, Madrid, Spain; 3 South Kyushu Livestock Veterinary Center, Kagoshima University, Soo, Japan; 4 Laboratory of Microbiology, Department of Disease Control, Faculty of Veterinary Medicine, Hokkaido University, Sapporo, Japan; 5 Global Station for Zoonosis Control, Global Institute for Collaborative Research and Education, Hokkaido University, Sapporo, Japan

**Keywords:** Ecological epidemiology, Ecological modelling, Invasive species, Microbial ecology, Population dynamics, Computational biology and bioinformatics, Ecology, Microbiology, Ecology, Environmental sciences, Diseases, Risk factors, Information technology, Statistics

## Abstract

Classical swine fever has been spreading across the country since its re-emergence in Japan in 2018. Gifu Prefecture has been working diligently to control the disease through the oral vaccine dissemination targeting wild boars. Although vaccines were sprayed at 14,000 locations between 2019 and 2020, vaccine ingestion by wild boars was only confirmed at 30% of the locations. Here, we predicted the vaccine ingestion rate at each point by Random Forest modeling based on vaccine dissemination data and created prediction surfaces for the probability of vaccine ingestion by wild boar using spatial interpolation techniques. Consequently, the distance from the vaccination point to the water source was the most important variable, followed by elevation, season, road density, and slope. The area under the curve, model accuracy, sensitivity, and specificity for model evaluation were 0.760, 0.678, 0.661, and 0.685, respectively. Areas with high probability of wild boar vaccination were predicted in northern, eastern, and western part of Gifu. Leave-One-Out Cross Validation results showed that Kriging approach was more accurate than the Inverse distance weighting method. We emphasize that effective vaccination strategies based on epidemiological data are essential for disease control and that our proposed tool is also applicable for other wildlife diseases.

## Introduction

Classical swine fever (CSF), caused by classical swine fever virus of the genus *Pestivirus* of the family Flaviviridae, is an infectious viral disease of domestic and wild pigs. The disease is considered one of the most important transboundary swine diseases, along with ASF and Foot and Mouth Disease, because of its potential to severely impact the swine industry. The most common mode of transmission is through direct contact between a healthy susceptible host and infected animals^1^. The disease can also be spread through the discharge of infected animals and contaminated pork products, thus contaminated food residues, vehicles, and clothing are important sources of indirect transmission routes^2^. The disease has acute and chronic forms, ranging from severe with high mortality to mild or no symptoms. Clinical signs are known to be very similar to those of African swine fever (ASF)^3^. Geographically, CSF is distributed in parts of Latin America, Europe, Asia, and Africa. According to the World Organisation for Animal Health (WOAH), vaccination can prevent the spread of the disease in areas where the disease is endemic^1^.

Japan has successfully eradicated CSF through vaccination efforts in the twentieth century, owing to the availability of highly effective vaccines^4,5^. However, since the re-emergence of CSF in 2018, the infected area has expanded, notably due to large outbreaks in wild boar populations along with sporadic outbreaks at pig farms^6^. The current CSF virus epidemic strain in Japan is considered moderately virulent, with a mix of individuals dying from infection and those surviving^7^, contributing to the large-scale expansion of infection^8^. Vaccination of domestic pigs and wild boars began in October and March 2019, respectively^9^. While vaccination is encouraged across most regions in Japan, the lack of control of the epidemic among wild boars remains a major challenge. As of August 31, 2023, CSF infections have been reported in 24 prefectures for domestic pigs and 35 prefectures for wild boars, covering all regions except Hokkaido (Honshu, Shikoku, Kyushu, and Okinawa)^10^.

Wild boars, which are omnivorous and have a wide range of food choices, are widely distributed in Japan and appear in rice paddies and farmlands adjacent to their habitat^11,12^, and are known to selectively visit broadleaf forests, abandoned farmlands, and bamboo forests in particular^13,14^. In addition to digging up the ground and feeding on plant roots and rhizomes, they also eat acorns and prey on insects and reptiles. They feed on bamboo shoots in spring, rice in summer, hard fruits in autumn, and an increasing proportion of plant roots and tubers in winter^14–16^. A study by the Ministry of the Environment found that the distribution range of wild boars has expanded about 1.9 times over the past 40 years^17^, indicating that CSF could further expand and have a more serious impact on the Japanese swine industry.

Vaccination is a key preventative measure for both individuals and communities against infectious diseases, crucial for preventing future infections and controlling disease spread. In wildlife populations where interventionist management is more challenging compared to livestock, the introduction of oral vaccines has been recognized as a key strategy for controlling diseases^18^. Gifu Prefecture, where there have been ongoing outbreaks since the first introduction, is one of the municipalities that has been working diligently to control the disease through oral vaccine dissemination for wild boars. Based on the insights of local hunters and wild boar experts, the oral vaccine was dispersed at a total of about 14,000 sites over three seasons each year from 2019 to 2020. At each site, 10 or 20 vaccines were spread and buried in holes dug about 10–15 cm deep. In each hole, one or two vaccines were buried together with lure food consisting of compound feed and pressed corn, and the surface of the hole was covered with lure food. To measure the effectiveness of the vaccination application, the remaining vaccine or vaccine packet was collected 5 days after application and the presence or absence of feeding by wild boar was determined based on the shape of the remaining vaccine packet^19^. Consequently, vaccine ingestion by wild boars was confirmed in approximately 30% of the dispersal sites^20^. The expansion of infected areas and the prolonged infection period will burden the economy, as will the cost of control measures. Therefore, the development of effective vaccination strategies is an urgent priority in the current epidemic situation.

In the previous study, we performed a generalized linear mixed model (GLMM) analysis based on data from sensor cameras installed at approximately 10% of all vaccine dispersal points to identify areas where wild boar was most likely to appear. We found a positive correlation between the emergence of other wildlife (raccoon, raccoon dog, and crow) and the emergence of wild boar^20^. In addition, road density and vegetation were also estimated to influence wild boar emergence. However, areas of high wild boar emergence may not coincide with areas of high vaccine feeding. Estimation of areas with high potential for vaccine feeding by wild boars based on accumulated large-scale data would be useful for an efficient selection of vaccine application sites.

Machine learning algorithms can analyze large, complex data sets and identify patterns and trends that are difficult to be detected by humans. For this reason, an increasing number of studies in recent years have applied them to predict infectious disease outbreaks^21^. Random Forest^22^ is one of the most commonly used and most powerful machine learning techniques or this purpose^21^. It is an algorithm that combines several randomized decision trees and aggregates the predictions by averaging^23^.

In this study, we first predicted the wild boar vaccine ingestion rate at each vaccine dissemination site based on a Random Forest model. The obtained results were then combined with spatial interpolation techniques to output a prediction surface showing the vaccine ingestion probability of wild boars in Gifu Prefecture. Effective vaccination strategies for wild animals can be summarized in two aspects: high efficacy of the vaccine itself and efficient vaccine delivery to each individual. This study aimed to contribute to the control of CSF in wild boars by focusing on the latter.

## Materials and methods

### Characteristics of the study area and data collection: Gifu Prefecture

**Figure 1:**
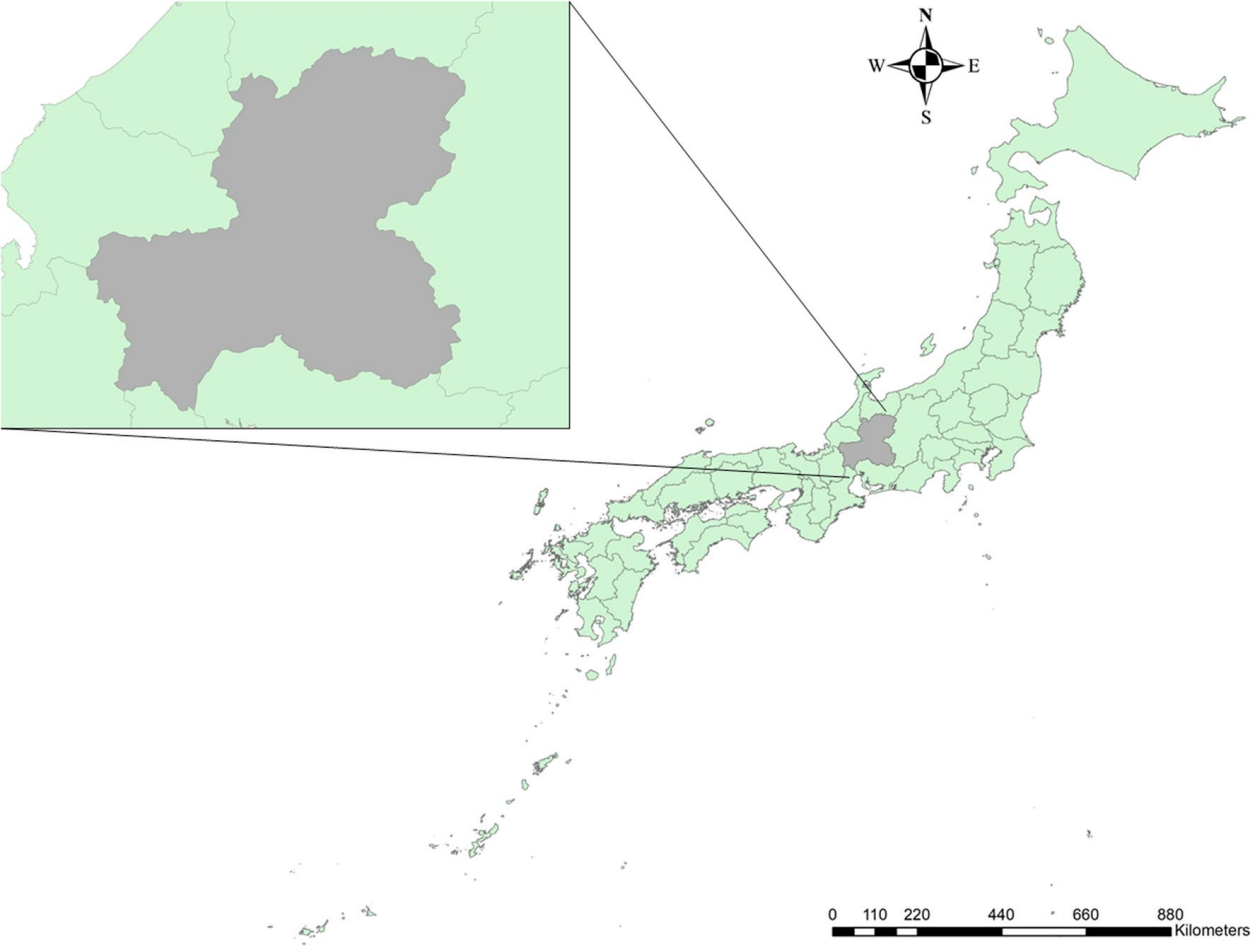
Location of Gifu Prefecture. Gifu is located in the center of Japan. The map was depicted in ArcGIS 10.8.1 (Esri).

The selected study area, Gifu Prefecture, is located in central Japan (Fig. 1). This is the area where the re-emergence of CSF was reported in 2018. Forests cover 82% of the prefectural land, and the northern part of the prefecture is covered with mountains over 3000 m in elevation, while the southern part is covered with plains. Because of the large difference in elevation and climate within the prefecture, a wide variety of plant communities can be observed, including evergreen broad-leaved forests in the warm temperate zone, deciduous broad-leaved forests in the cool temperate zone, coniferous forests in the subarctic zone, and alpine plants^24–26^. The estimated wild boar population in the prefecture was about 16,000 at the end of March 2019, and then halved for a time due to the CSF epidemic. However, it has recently been gradually recovering, and as of the end of March 2022, approximately 17,600 animals are estimated to be present in a wide range of areas^27^.

The present study was conducted based on CSF oral vaccine dissemination data administered by Gifu Prefecture from March 24, 2019 to November 12, 2020. These data are not available to the public and were provided by Gifu Prefecture on a conditional basis. In 2019, oral vaccine dissemination was performed six times, twice each in spring (March and May), summer (July and August), and winter (December 2019 and February 2020). In 2020, the vaccine was dispersed once each in spring (April), summer (June), and autumn (October–November). During this period, oral vaccine was disseminated to a total of 14,131 locations in Gifu Prefecture. Of these, data for 10,879 locations available as Excel data were used in this study. These data included geographic XY coordinates of distribution points, dates of vaccine dissemination and collection, number of vaccines disseminated, number of vaccines remaining at the time of collection, number of vaccines with evidence of feeding by wild boars, number of vaccines with evidence of bites by other animals, and presence of traces of wild boars in the surrounding area.

### Classification tree random forest: variable selection

Random Forest is an ensemble learning algorithm that integrates multiple decision trees. Each tree is trained on a random subset of the data, allowing the model to capture a broad spectrum of data characteristics. Predictions from individual trees are aggregated, typically through majority voting for classification or averaging for regression, to produce the final output. This approach effectively reduces overfitting, making Random Forests well-suited for handling complex datasets with numerous input variables^23^. To perform classification tree-based Random Forest in the R programming environment, we used the *ranger* package, which is particularly suitable for high-dimensional data and can be implemented at high speed^28^.

The response variable was set as "vaccine ingestion by wild boars at each oral vaccine application site (1 for ingestion, 0 for non-ingestion). Although the number of vaccines ingested by wild boars has been quantitatively recorded, the possibility of duplicate counts by the same individual cannot be excluded. The following explanatory variables were selected based on the ecological characteristics of wild boars and authors’ previous experience with wild boar-related studies: season of vaccination (spring, summer, autumn, and winter), presence or absence of vaccine feeding by other animals, presence or absence of wild boar traces in the surrounding area, elevation, slope, road density, distance from water sources, human footprint (a measure of human activity area indicator), vegetation, and bioclimatic factors (Table 1). Vaccine feeding status is presumably influenced by the behavioral patterns and available food resources of wild boars, which vary by season. The variable of vaccine dissemination season, a categorical variable, was subjected to a label encoding process. Our previous study found a correlation between the emergence of other animals and wild boar^20^, and therefore it may also influence the vaccine uptake rate of wild boar. The vaccine ingestion variable by other animals was treated as binary data for the same reasons as in the response variable setting. If vaccine dissemination sites overlap with wild boar activity zones, the uptake rate is expected to be higher. Therefore, the presence or absence of wild boar traces in the surrounding area may be related to vaccine uptake. In a previous study, elevation and slope were identified as factors affecting wild boar habitat^29^, and these factors may have influenced the response variable in Gifu, where there is a large elevation difference. These data were downloaded as shapefiles from the National Land Numerical Information Download Service (NLNIDS) provided by the Ministry of Land, Infrastructure, Transport and Tourism^30^. The information corresponding to the distribution points was then extracted using the Intersect tool in ArcGIS 10.8.1 (Esri)^31^. As wild boars are inherently cautious animals^32^, areas of human activity and road density can be associated with vaccine feeding rates. Human Footprint data representing areas of human activity were downloaded from the SEDAC database^33^, and values for each distribution point were obtained using the Extract Multi Values to Points tool in ArcGIS^34^. Road density data were obtained as shapefiles from NLNIDS and values were extracted for each location using the Intersect tool in ArcGIS. Water is an essential element for sustaining life and is closely related to feeding behavior. Therefore, distance from the water source is assumed to have some influence on the response variable. Here, rivers, streams, and lakes were defined as water sources, and geographic information was downloaded from NLNIDS. The Euclidean distance from each vaccine dispersal point to the nearest water source was calculated using the Near tool in ArcGIS^35^. Vegetation and bioclimatic factors are frequently used in species distribution models and are often included in studies to predict ASF epidemics in wild boars and to elucidate risk factors^36–39^. Indeed, these factors are assumed to have a significant influence on the behavioral patterns of wild boars and the availability of food resources. In this study, vegetation information, including 58 vegetation classifications, was downloaded as shapefiles from the Biodiversity Center of Japan^40^. To fit these data quantitatively to each vaccine distribution point, Gifu Prefecture was first divided into a 1 km × 1 km grid using the Fishnet tool in ArcGIS^41^. The Tabulate Intersection tool in ArcGIS and the pivot function in Excel were then applied to calculate the percentage of each vegetation type in each grid^42^. Finally, the Extract values to points tool was used to link the data for each dispersal point to the percentage of vegetation composition in the grid to which it belongs. To avoid model complexity, an approach was applied in which only the main vegetation types that comprise Gifu Prefecture were retained as explanatory variables. Specifically, vegetation types that do not exist in Gifu Prefecture and vegetation types with less than 1% of the total number of dispersal points to which they belong were eliminated in this process. We downloaded the standard 19 bioclimatic variables for WorldClim version 2, representing the average values from 1970 to 2000. Based on our knowledge and experience, the following variables were selected for our analysis. bio4 (seasonality of temperature), bio5 (maximum temperature in warm months), bio7 (annual range of temperature), bio8 (average temperature in wettest quarter), bio13 (precipitation in wettest month), bio15 (precipitation in driest quarter), bio16 (precipitation in driest quarter), bio17 (precipitation in driest quarter), bio18 (precipitation in the warmest quarter), bio19 (precipitation in the coldest quarter)^43^. The geographic dataset used was a 1 km × 1 km spatial resolution in ArcGIS.

**Table 1:** List of variables included in Random Forest model.

Explanatory variables	Original data type
Vaccine-disseminated season	Point
Vaccine ingestion by other animals	Point
Presence or absence of wild boar traces in the surrounding area	Point
Distance to the water source	Point
Altitude	Polygon
Slope	Polygon
Road density	polygon
Human Footprint	Raster
Vegetation types	Raster
bio4(Temperature Seasonality)	Raster
bio5(Max Temperature of Warmest Month)	Raster
bio7(Temperature Annual Range)	Raster
bio8(Mean Temperature of Wettest Quarter)	Raster
bio13(Precipitation of Wettest Month)	Raster
bio15(Precipitation Seasonality)	Raster
bio16(Precipitation of Wettest Quarter)	Raster
bio17(Precipitation of Driest Quarter)	Raster
bio18(Precipitation of Warmest Quarter)	Raster
bio19(Precipitation of Coldest Quarter)	Raster

### Classification tree random forests: model implementation

Initially, the number of samples with a response variable of 0 was adjusted to equal the number of samples with a response variable of 1, using the *sample_n* function from *dplyr* package in R^44^. Then, multicollinearity among the explanatory variables was checked with respect to the Pearson correlation coefficient of the *cor* function in R. Only one of the variable pairs with a correlation greater than 0.6 was retained, based on the authors' knowledge and experience.

Random forests were classified into a supervised learning algorithm, thus 80% and 20% of the data were assigned as training and test data, respectively. The algorithm is known to give good results with default settings, but performance can be improved by adjusting the hyperparameter values^30^. To evaluate all possible combinations of parameter space based on RMSE (Root Mean Squared Error), various combinations of hyperparameter values were attempted using the *expand.grid* function (Table 2).

**Table 2:** Hyperparameter adjustment of random forest model.

Hyperparameters	Test conditions
Number of features considered at each split (mtry)	Number of variables × (0.05, 0.15, 0.25, 0.33, 0.4)
Minimum node size	1, 3, 5, 10, 15
Replace	True or False
Sample fraction	0.5, 0.55, 0.6, 0.65, 0.7, 0.75, 0.8
Number of trees	250, 300, 350, 400, 450, 500, 550, 600, 800, 1000, 2000

The model was trained using the optimal hyperparameter combination with the lowest RMSE, and the importance of the variables was quantified by the *vip* function from the *vip* package in R^30^. Predictive performance was evaluated using the measure of area under the curve (AUC) of the receiver operating characteristic (ROC) curve on the test dataset with the *pROC* package *auc* function in R^45^. The sensitivity and specificity of the model were further assessed using the *confusionMatrix* function of the *caret* package in R^46^.

### Spatial interpolation: inverse distance weighting (IDW) and Kriging

According to our previous work, two spatial interpolation methods (IDW and Kriging) were applied for interpolating spatial data between wild boar vaccine intake probabilities at each location given by a Random Forest model^40^. The IDW method is called deterministic interpolation, where ambient measurements determine the smoothness of the output surfaces and directly affect the results^43^. This approach assumes that values closer to one value being interpolated are more relevant than values farther away, and the values are obtained by a weighted average with the inverse of the distance as the weight. All calculations were performed with the *idw* function of the *gstat* package in R^43^, and the predicted surface output was performed in ArcGIS 10.8.1.

Kriging is a multi-step approach based on a geostatistical method that considers spatial autocorrelation. It evaluates the characteristics of the spatial probability field based on measured data and performs spatial interpolation from observed values considering the estimated spatial probability field^43^. At a specific distance (range), autocorrelations between data points become independent, meaning that when their variation stabilizes (sill), spatial autocorrelation among the data points' proximities ceases to exist. The following calculations were performed to estimate parameters (sill, range, nugget) representing spatial autocorrelation and range of variance. First, a variogram cloud was created using the *variogram* function of the *gstat* package to evaluate sample value pairs. Next, an experimental variogram was created using the *variogram* function. The nugget represents the variogram's y-intercept, attributed to measurement errors or spatial variations occurring at distances shorter than the sampling interval. It signifies the smallest scale of spatial variation that the study's sampling approach can detect. In the step of generating the variogram model using the *vgm* function, the best model was selected from the three models “Sph”, “Exp”, and “Gau” by the *fit.variogram* function. Based on the obtained model and the default values of the parameters (sill, range, nugget), both the experimental variogram and the model were overlaid and plotted to evaluate the initial model. The model was then fitted using one of the fit criteria, and ordinary kriging was performed with the *krige* function of the same package. Predictive surface mapping was performed on ArcGIS.

The Leave-One-Out Cross Validation method^43^ was applied to evaluate the prediction accuracy of the two spatial interpolation methods, and the final results were obtained as a table containing the observed and predicted values for all points. Validation was performed for each method using the *gstat.cv* function from the *gstat* package. The columns of predicted and observed values in the resulting table were used to calculate the RMSE^43^.

## Results

### Classification tree random forest: variable selection

Of the 58 vegetation taxa in the vegetation variable selection process, 18 were chosen as individual explanatory variables (Supplementary Table 1). A total of 10,245 vaccine spread sites remained throughout this process and were used in subsequent Random Forest implementations. Since the test data set contained 2227 samples with response variable of 1, an equivalent number of samples with a response variable of 0 were randomly selected by the *sample_n* function in R and used for further analysis. Of the variable combinations that had Pearson's correlation coefficients greater than 0.6, bio4, 5, 8, and 18 were excluded as candidate explanatory variables due to multicollinearity.

### Classification tree random forest: model implementation

A grid search resulted in an optimal Random Forest model based on the following combination of hyperparameter values with the lowest OOB (Out of Bag) RMSE of 0.419; mtry: 9, minimum node size: 15, replace: False, sample fraction: 0.55, number of trees: 2000 sample fraction: 0.55, number of trees: 2000.

**Figure 2:**
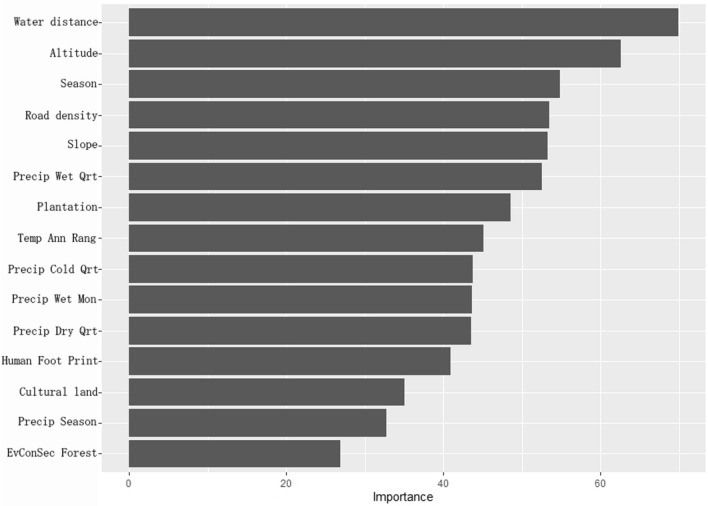
Importance rank of explanatory variables. Variable importance scores of the top 15 features from the random forest model are visualized using the *vip* package in R.

**Figure 3:**
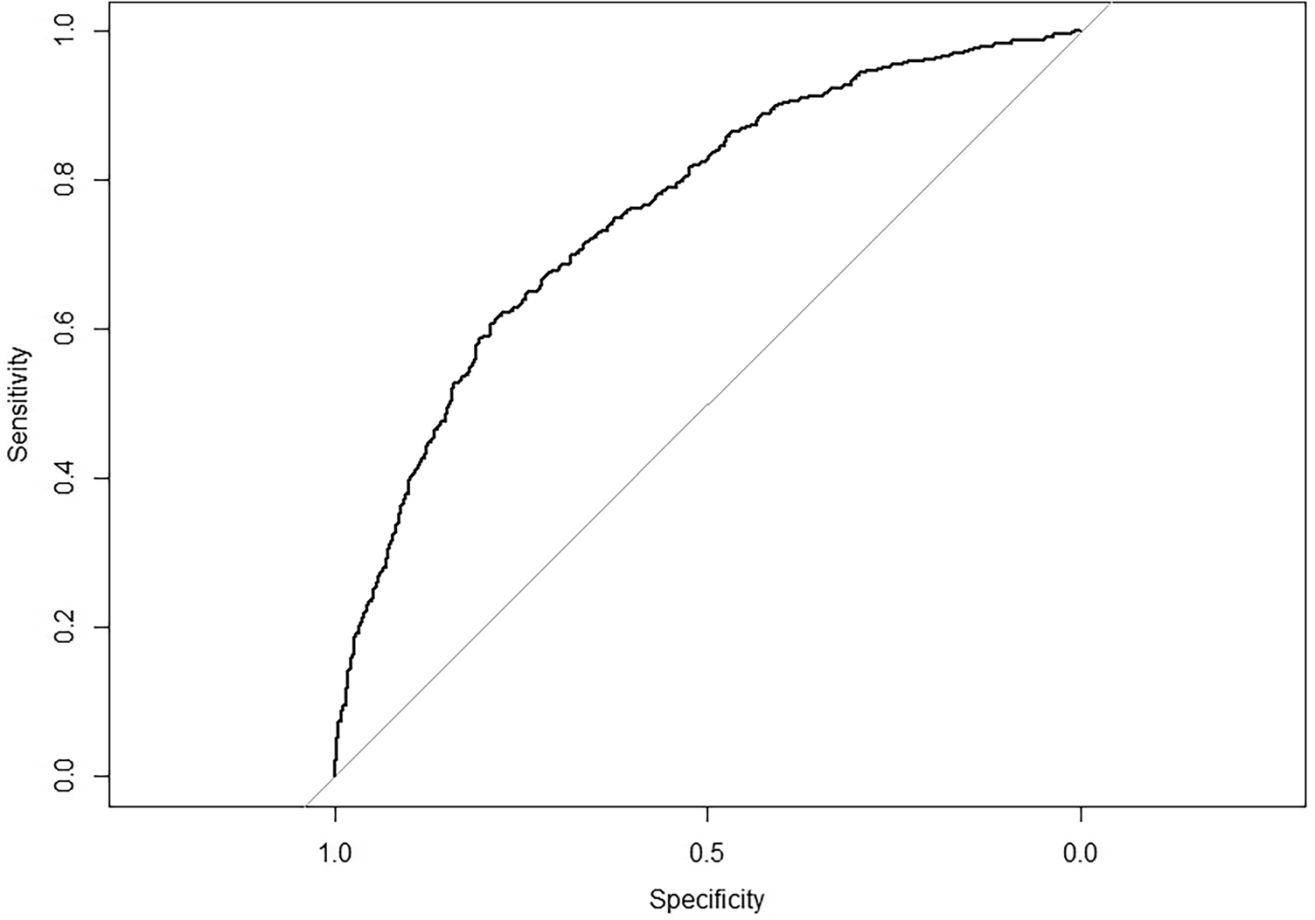
Model evaluation by ROC curve. The ROC curve of the model was plotted using the *roc* and *auc* functions of the *pROC* package on R programming and AUC = 0.760 was calculated.

The top 15 variables of importance in this model were shown in Fig. 2. Distance from the vaccine distribution point to the water source was the most important variable, followed by elevation, season, road density, and slope (Fig. 2). The AUC for assessing model performance was 0.760 (Model accuracy = 0.678, sensitivity = 0.661, specificity = 0.685) (Fig. 3).

### Spatial interpolation: inverse distance weighting (IDW) and Kriging

**Figure 4:**
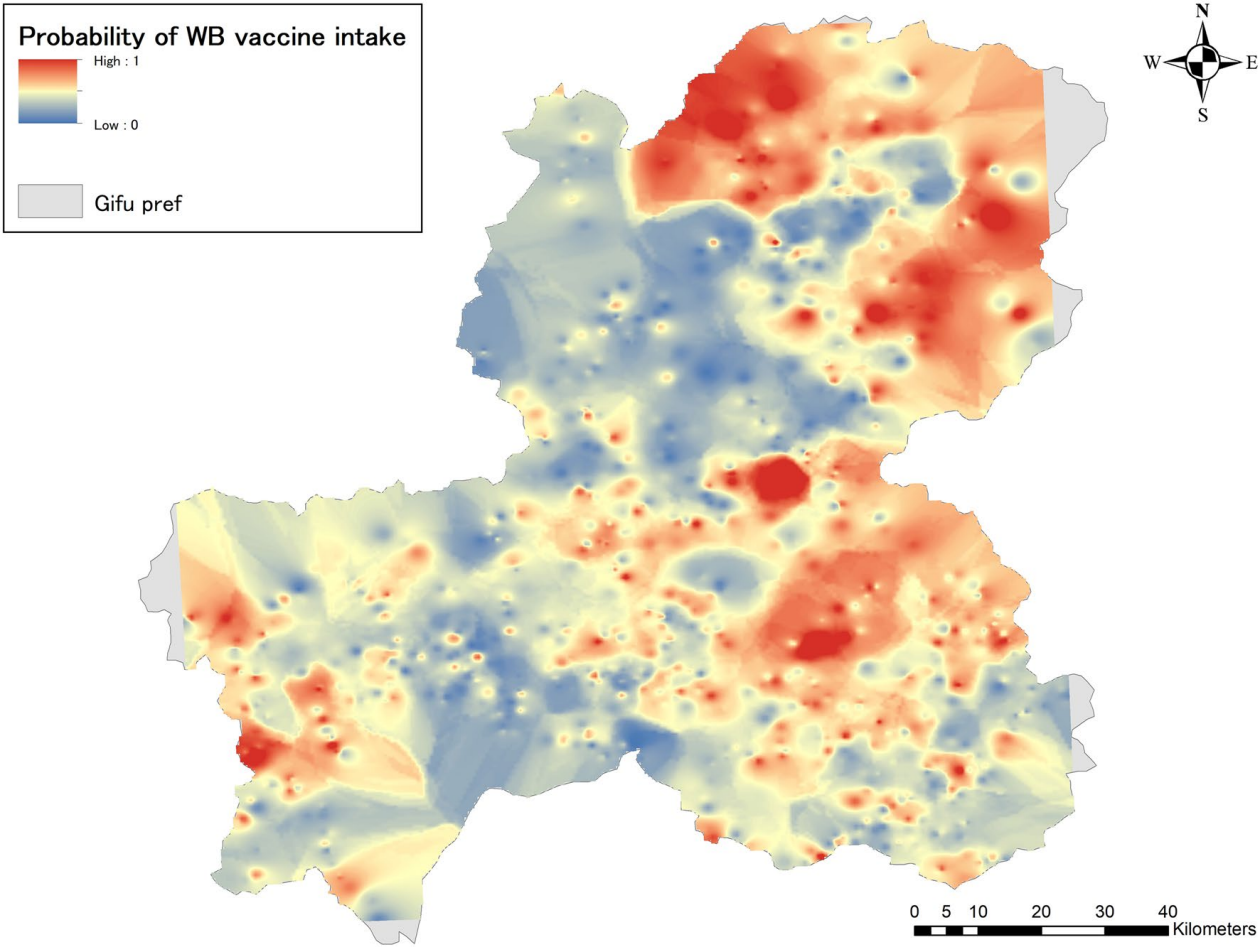
Probability of wild boar vaccine intake by IDW (Inverse distance weighting) method. Surfaces of wild boar oral vaccine intake probability in Gifu Prefecture was created using the IDW method and depicted in ArcGIS. The probabilities take the range 0–1, with blue areas showing a lower probability of intake and red areas showing a higher probability of intake. The gray-colored areas represent regions within Gifu Prefecture that couldn’t be analyzed using spatial interpolation due to the absence of nearby vaccine distribution points.

Wild boar vaccine intake probability maps interpolated by the IDW method were depicted on ArcGIS 10.8.1. Areas of high probability were identified from northeastern to southeastern Gifu Prefecture and also in the western part of the prefecture (Fig. 4).

**Figure 5:**
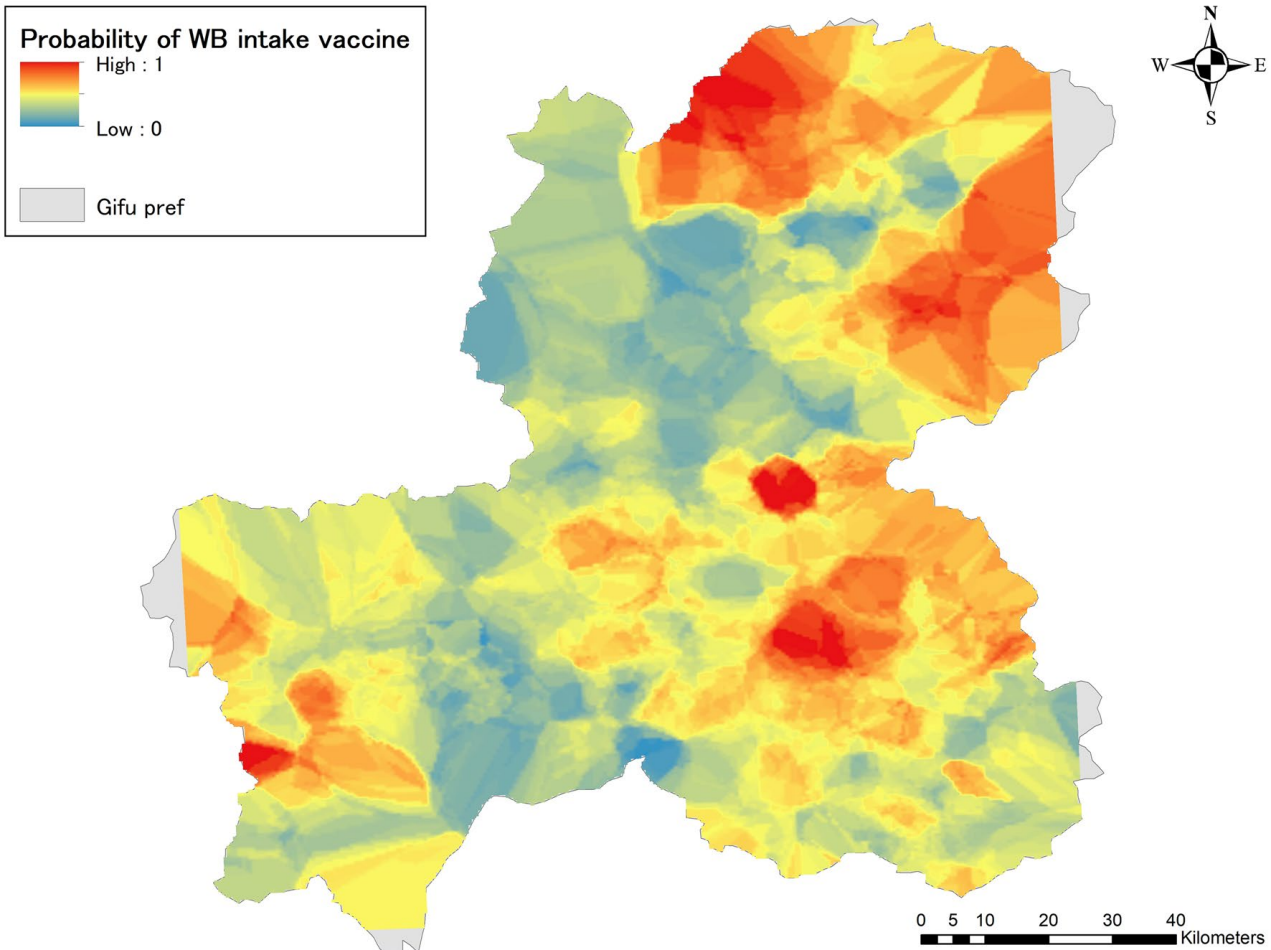
Probability of wild boar vaccine intake by Kriging method. Surfaces of wild boar oral vaccine intake probability for Gifu Prefecture was created using the Kriging method and depicted in ArcGIS. Probabilities take the range 0–1, with blue areas indicating a lower probability of intake and red areas indicating a higher probability of intake. The gray-colored areas represent regions within Gifu Prefecture that couldn’t be analyzed using spatial interpolation due to the absence of nearby vaccine distribution points.

In the Kriging method, the empirical semivariogram was approximated by a model, and “Sph” was chosen as the best model, with values of 0.133, 0.189, and 35.68 for Nugget, Sill, and Range, respectively. Using these values, a predictive surface of wild boar vaccine intake probability in Gifu Prefecture was depicted (Fig. 5). The overall spatial distribution of probability is similar to the IDW results, but the high probability areas tend to be more aggregated compared to the IDW results, where high probability areas are scattered throughout the entire area.

Finally, the prediction accuracy of the IDW and Kriging methods was evaluated using Leave-One-Out Cross Validation. The results showed that the RMSE for IDW and Kriging were 0.179 and 0.168, respectively, indicating that Kriging was more accurate in prediction in this analysis.

## Discussion

In this study, we employed machine learning models on large-scale oral vaccine distribution data to identify key factors influencing wild boar vaccine uptake. Spatial interpolation techniques were integrated to predict areas of high vaccine uptake probability, aiming to improve CSF management strategies among wild boars. Although the Random Forest modeling achieved fairly good predictive accuracy^47^ with an AUC of 0.760, there is potential for improvement, especially in accurately reflecting wild boar population density and the human impact on vaccine distribution. The research underscores the necessity for effective vaccination strategies and the challenges in managing disease spread among wildlife. The comprehensive approach combining machine learning and spatial analysis offered significant insights for disease management in wild boar populations and the protection of the swine industry.

The results of the optimized random forest model identified important variables including distance to water sources, elevation, season, road density, and slope. Distance to the water source was chosen as the most important variable. This result seems logical given the relationship between food resources and water, which is essential for sustaining life. Wild boars should have a water source in the vicinity of their living area, which may make it easier for bait vaccines to be found as a food source when wild boars move around the area. Previous studies have reported that the distribution and abundance of resources significantly influence the movement patterns of wild boars. The presence of high-quality and abundant food and water sources tends to reduce the home range of wild boars. Conversely, poor nutritional conditions lead wild boars to move more extensively in search of food and water^48^. Elevation and slope would shape the flow of water and furthermore affect vegetation distribution and temperature changes. These variables were selected as important variables because they indirectly affect the ecology of wild boars^49^. The relationship between environmental factors and wild boars' behavioral patterns is frequently addressed in epidemiological studies identifying ASF risk factors. While interpreting behavior pattern changes due to infection requires caution, the close association between habitat-related factors, including water and vegetation, and wild boar behavior has been highlighted in various studies^50,51^. Seasonal changes would have affected the reproductive cycle and availability of food resources for wild boars, which would have led to changes in their feeding behavior patterns. Wild boars usually conceive once a year, either in spring or autumn, and give birth to about four young per litter^43^. As mentioned earlier, staple foods also vary by season, and the availability of these resources is not always constant. The bioclimatic factors listed as important variables (16,7,19,13,17,15) (see Table 1) have an indirect impact on life-sustaining resources (like water and food), vegetation, and the life cycle of wild boars. Thus, they would have been considered important. Vegetation types 54 (Plantation), 57 (cultural land), and 42 (Evergreen coniferous secondary forest) were identified as the important variables affecting the model in the present results. Cultural land has been identified as a preferred area for wild boars^43^. Cedar and cypress are planted in plantation in Gifu Prefecture, and the coniferous forest zone is one of the major vegetation types that comprise the forest area in the prefecture. It is interesting that coniferous forest zone was identified as an important variable in this study, even though broadleaf forest zone, which is considered favorable for wild boar, is also widely distributed. Road density and Human Footprint variables can be considered anthropogenic factors influencing wild boar habitat. According to the previous study, wild boar are less likely to appear in urban areas with high road density and high human activity^52^. Rather, wild boars tend to appear in areas where road density is not very dense and human activity is not frequent, i.e., areas with diverse landscapes are highly important, such as at the boundary between mountainous areas and human settlements, where they are more likely to forage for food^53^.

The results obtained in this Random Forest modeling (AUC of 0.760) were fairly good prediction accuracy. The study incorporates all currently available information based on previous findings. However, the sensitivity and specificity results obtained still suggest issues with prediction accuracy and aspects that need to be improved. A similar study conducted previously in Gifu Prefecture indicated a correlation between vaccine intake and population density^54^. Information on the density distribution of wild boars is expected to improve the prediction accuracy of this model, but challenges remain to reflect actual conditions. Detailed population density data would be difficult to be obtained as it is highly variable and heavily influenced by CSF epidemic status and depopulation control. There are also several aspects that are difficult to be quantified in the data because vaccine dissemination is conducted manually by human operators. Wild boars are generally very cautious and prefer to live in mountain forests where there is little human access. The smell and traces left by humans around the dissemination point during vaccine application operations may have an impact on the appearance of wild boars as well as on vaccine uptake. If this information can be quantified in some way, the prediction accuracy of the model will be improved. The wild boar trace variable was not selected as the top important variable in this analysis. Given that this work was done visually by field operators, the accuracy of the information is dependent on their experience and skill. A vaccine packet that has been chewed and has not retained its original shape is regarded as a feeding by wild boar. However, it is difficult to make an accurate evaluation based on this alone, and therefore, a certain degree of bias due to human factors cannot be excluded at the stage of response variable selection. A more reliable judgment can be made if the presence or absence of foraging behavior is confirmed. In our previous study, we found a significant positive correlation between the appearance rate of wild boars and that of other animals. Contrary to our assumption that these animals could be competitors for vaccine uptake by wild boars, it was not selected as a key variable in the model. Regardless of the presence or absence of vaccine ingestion by wild boars, vaccine ingestion or removal by other animals was documented at the majority of dispersal sites. This poses a major challenge for effective vaccine dissemination. Based on our previous results, it is highly likely that small and medium-sized animals such as raccoon dogs, raccoons, and crows are involved. As a countermeasure, Gifu Prefecture has taken steps to place stones at dispersal points that can only be moved by wild boars.

When the data contains information that can be attributed only to individual points, the application of spatial interpolation is useful for interpolating between points and creating prediction surfaces. In this respect, the current approach, combining machine learning techniques and spatial interpolation, is unique. Both two spatial interpolated wild boar oral vaccine intake probability maps estimated high probability areas in the northeastern and southeastern parts of Gifu Prefecture, as well as in the western region. The difference in output surface smoothness may be originated from algorithmic differences, but the spatial distribution of high probability areas in both maps is very similar. The results of the RMSE cross-validation test concluded that the prediction accuracy of Kriging was slightly higher, consistent with our previous study results^55^. As previously mentioned, the probability of wild boar appearances and vaccine feeding rates may not always coincide, suggesting external influences on their feeding habits besides individual factors. Consequently, the presence of wild boars does not guarantee vaccine intake. Environmental effects, along with the vaccine distribution technique and the characteristics of the vaccine itself, may also play significant roles^56^. Compared to the wild boar emergence map created in the previous study (Supplementary Fig. 1), both analyses observed high probability areas in the northern and southeastern parts of the prefecture. On the other hand, there are differences between the two analyses in the western and central parts of the prefecture. Although the data, methods, and objectives used are different and cannot be compared, analyzing the similarities and differences between the two studies may be useful in prioritizing vaccine application sites. For example, in the western part of the prefecture, the probability of wild boar emergence is not very high, but the oral vaccine uptake rate is relatively high. Conversely, the southeastern border area of the prefecture has a high probability of wild boar emergence but not a very high probability of vaccine uptake, so it will be necessary to rethink measures in terms of cost-effectiveness. The results obtained here should be shared with experts familiar with local geography and wild boar ecology and utilized effectively for implementing countermeasures.

Several studies have already been conducted on the development of effective vaccination strategies for wild boars in Japan, including our previous study. Ikeda et al.^57^ identified factors influencing wild boar vaccine uptake rates from biological, environmental, and geographical perspectives using spatial Bayesian generalized linear model. Endo et al.^53^ used a generalized linear mixed model to investigate landscape factors correlated with vaccine uptake. These studies analyzed causal relationships modeled by regression analysis. On the other hand, machine learning can be a powerful tool when high predictive accuracy is required, and its use in veterinary public health surveillance has expanded in recent years^58^. Our study focused on a specific local authority holding more than 10,000 data accumulated over several years. Additional incorporation of spatial interpolation allows the results to be shared visually, facilitating the prioritization of vaccination points. In the same region, changes in antibody prevalence against CSF due to vaccine campaigns in wild boar populations and the amount of vaccination required to eliminate CSF have been estimated based on mathematical modeling^59^. Accordingly, both studies can be taken into account for more appropriate selection of vaccination strategy.

Although this study focused on oral vaccination of wild boars against CSF, the proposed tool may also be useful in controlling other diseases. As the prevalence of CSF in Japan has shown, control of the disease in wildlife is very difficult, even when an effective vaccine is available. A similar disease, ASF, has recently caused a large-scale epidemic on a global level. In Europe, the disease continues to spread mainly among wild boars, and in Asia, both domestic pigs and wild boars have suffered from ASF. It is important to remember that early disease control requires effective vaccination strategies based on epidemiological studies as well as vaccine efficacy.

### Supplementary Information

Supplementary Figure 1.

Supplementary Table 1.

## Data Availability

The data that support the findings of this study are available from Gifu prefecture, but restrictions apply to the availability of these data, which were used under license for the current study, and so are not publicly available. Data are however available from the authors upon reasonable request and with permission of Gifu prefecture.
