# Supplementary material for: Application of machine learning with large-scale data for an effective vaccination against classical swine fever for wild boar in Japan
Source: Sci Rep. 2024 Mar 4;14:5312. doi: 10.1038/s41598-024-55828-6 (PMC10912211; doi:10.1038/s41598-024-55828-6)
Supplement: Supplementary file 1 — Supplementary Figure 1. [file 41598_2024_55828_MOESM1_ESM.docx]

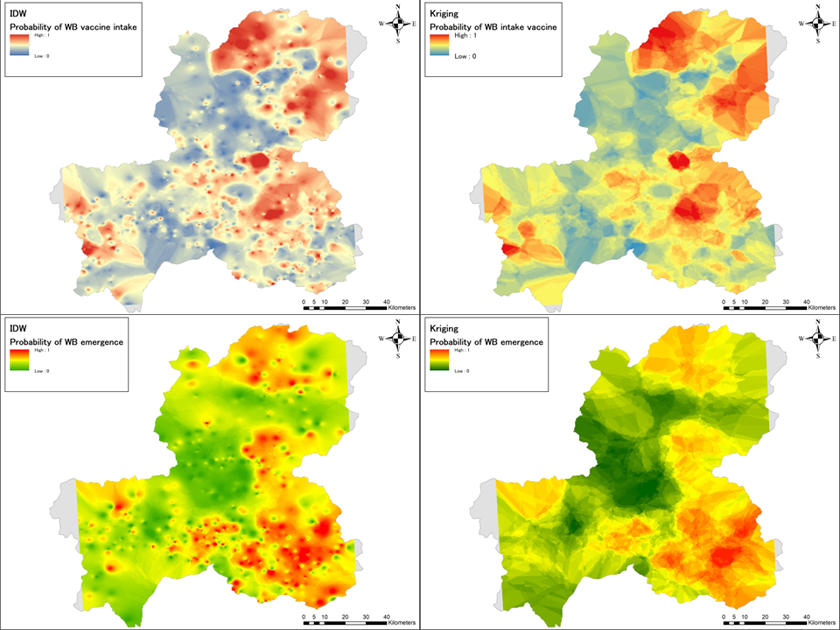


**Supplementary Figure 1. Visual comparison of wild boar emergence and vaccine uptake rates by spatial interpolation method.** Top left and right figures are wild boar oral vaccine ingestion probability maps based on Random Forest modeling interpolated by IDW and Kriging, respectively. Lower left and lower right figures are wild boar appearance probability maps based on camera data applying GLMM interpolated by IDW and Kriging, respectively (see previous study results) ^20^. The gray-colored areas represent regions within Gifu Prefecture that couldn’t be analyzed using spatial interpolation due to the absence of nearby vaccine distribution points.
